# Design and Performance Analysis of an RIS-Empowered RM-DCSK System for Wireless Powered Communication

**DOI:** 10.3390/e28030300

**Published:** 2026-03-05

**Authors:** Fang Liu, Junjun Ma, Qihao Yu

**Affiliations:** 1 School of Information Science and Engineering, Shenyang Ligong University, Shenyang 110159, China; g08852070@gmail.com; 2 School of Equipment Engineering, Shenyang Ligong University, Shenyang 110159, China; yuqihao@sylu.edu.cn

**Keywords:** chaotic communication, wireless powered communication, reference-modulated differential chaos shift keying, reconfigurable intelligent surface, bit error rate

## Abstract

This paper proposed a reconfigurable intelligent surface (RIS)-empowered reference-modulated differential chaos shift keying (RM-DCSK) wireless powered communication (WPC) system. As a noncoherent chaotic communication scheme, the proposed system exploits the reference reuse property of RM-DCSK, where the reference signal simultaneously carries data information, thereby improving spectral efficiency while maintaining noncoherent and channel-estimation-free reception with low receiver circuit complexity. Furthermore, RIS is utilized to reconfigure the propagation environment and mitigate the path loss effect of WPC links. At the user equipment (UE), a harvest–store–use (HSU) energy harvesting and finite-buffer model is developed, and a threshold-based on/off transmission policy is adopted to enable sustainable uplink transmission. To quantify the gain of energy buffering and management, a bufferless baseline system is further established. Closed-form bit error rate (BER) expressions are obtained under multi-path Rayleigh fading channels for both the proposed RIS-RM-DCSK-WPC system and bufferless baseline system. Finally, simulation results validate the analysis and demonstrate that the proposed system achieves superior BER performance compared with representative benchmarks, including existing RIS-aided DCSK-WPC, RM-DCSK-WPC, and bufferless RIS-RM-DCSK-WPC systems.

## 1. Introduction

Chaotic signals possess unique characteristics including broadband spectrum, noise-like waveforms, sensitivity to initial conditions, and inherent aperiodicity [1,2,3], which make the chaotic signals attractive for secure and interference-resistant wireless communication. Notably, differential chaos shift keying (DCSK) has been extensively investigated as a noncoherent chaos-based communication scheme. Owing to its distinct advantages, including the absence of a chaotic synchronizer, low implementation complexity, enhanced physical-layer security, and strong resistance to multi-path interference [4,5,6], DCSK has attracted considerable attention for applications in complex wireless environments.

In conventional DCSK [7], the reference segment occupies half of the duration of the symbol, which limits the time available for effective information transmission and reduces spectral efficiency. This inherent limitation results in poor energy utilization and a limited achievable data rate, posing a challenge to meet the requirements for high-speed communications. To overcome the problem, various DCSK variants have been developed. For instance, a high-efficiency DCSK (HE-DCSK) was reported in [8], which doubles the data rate by recycling reference samples. Similarly, a reference modulated DCSK (RM-DCSK) [9] was developed to enable the reference signal itself to convey information, thereby significantly enhancing spectral efficiency while maintaining low complexity. In addition, other enhanced DCSK architectures have been explored, including multi-carrier DCSK (MC-DCSK) [10], short reference DCSK (SR-DCSK) [11], and carrier index modulation DCSK [12], and other variants have been proposed successively. By reducing reference overhead or exploiting additional transmission dimensions, these schemes alleviate the inherent efficiency limitations of conventional DCSK and facilitate higher-rate noncoherent communications.

However, energy constraints remain a critical challenge for IoT deployments, despite the significant spectral efficiency enhancements achieved by the above schemes. Conventional battery-powered solutions require periodic battery replacement, which is costly and sometimes impractical when the equipment are deployed in hazardous environments or embedded within the human body [13]. To address these energy constraints, a variety of energy harvesting techniques have been extensively investigated. Conventional ambient energy sources (e.g., solar, wind, and thermal energy) provide renewable power but suffer from intermittency and unpredictability due to their strong dependence on environmental conditions. In contrast, radio frequency energy harvesting (RF-EH) technology provides a practical means of supplying energy by converting RF signals into usable power, offering greater stability and controllability than conventional ambient energy sources. Over the past decade, the integration of simultaneous wireless information and power transfer (SWIPT) with DCSK has been extensively investigated. For example, an SR-DCSK-SWIPT scheme was reported in [14], where a shortened chaotic reference segment is employed to improve transmission efficiency while enabling energy harvesting. In [15], a CI-DCSK-SWIPT system was proposed, in which a time-switching (TS) protocol is employed to divide the transmitted signal into two consecutive phases, with the first phase dedicated to energy harvesting while the second is utilized for information decoding. Furthermore, the performance of DCSK-SWIPT has been validated in buffered relay systems [16] and two-way relay systems [17], demonstrating that such integration can further enhance system performance. However, these DCSK-SWIPT schemes primarily focus on simultaneous energy and information transmission in the downlink. In [18], the wireless powered communication (WPC) architecture was introduced to enable wireless devices to utilize harvested energy for uplink data transmission, which has since attracted considerable research attention [19,20,21].

To further alleviate severe path loss in wireless propagation environments and improve the information transmission quality and energy harvesting efficiency of DCSK-SWIPT systems, reconfigurable intelligent surface (RIS)-aided wireless communications have recently attracted significant attention as a promising solution for energy-efficient green wireless communications [22]. An RIS comprises an array of numerous passive reflecting elements, whose reflection coefficients can be configured in phase or amplitude to manipulate incident electromagnetic waves. By reconfiguring the propagation environment in a programmable manner, RIS can meet diverse service requirements, including improved spectral and energy efficiency, extended coverage, and enhanced link reliability [23,24,25]. Motivated by these advantages, RIS-aided DCSK has recently emerged as an active research topic. An RIS-aided DCSK has been proposed in [26], demonstrating that placing an RIS near the transmitter can boost the received SNR and thus significantly lower the BER, and [27] further studied an RIS-aided dual-mode DCSK (DM-DCSK) that increases the number of conveyed bits while achieving favorable BER performance over Rayleigh fading channels. In addition, in [28], an RIS-assisted FM-DCSK-SWIPT scheme was proposed to jointly facilitate signal enhancement and energy delivery. For the WPC architecture, [29] studied an RIS-aided DCSK system equipped with energy buffering, and demonstrated that energy storage can improve system stability. In [30], an RIS-aided short-reference DCSK-WPC scheme was developed to boost transmission efficiency by reducing the reference segment length.

Although existing schemes can alleviate the energy supply issue of wireless devices to a certain extent, they are still constrained by limited spectral efficiency and energy harvesting efficiency, which makes it difficult to support high-data-rate and low-power wireless communications. With the motivation mentioned above, an RIS-RM-DCSK-WPC system is proposed in this paper. Unlike conventional DCSK-SWIPT schemes that primarily focus on downlink performance optimization, the proposed scheme aims to boost uplink transmission reliability for energy-limited devices. The main contributions of this paper are summarized as follows:

1. An RIS-RM-DCSK-WPC system for energy-constrained uplink data backhaul is proposed. The system exploits the reference reuse property of RM-DCSK, where the reference signal simultaneously carries data information, which leads to an enhancement in system efficiency. Furthermore, an energy harvesting and buffer management strategy based on the HSU mechanism is developed at the user side. Through appropriate time slot partitioning and energy scheduling, energy self-sustainability and stable uplink transmission of energy-constrained devices can be achieved. Additionally, the RIS is introduced to dynamically reconfigure the wireless propagation environment, thereby effectively mitigating the double path loss effect inherent in WPC links.
2. A comprehensive analytical framework is developed to characterize the proposed RIS-RM-DCSK-WPC system. Moreover, to explicitly quantify the benefit of energy buffering and management, a bufferless RIS-RM-DCSK-WPC baseline system is constructed. Closed-form BER expressions are derived for both the proposed scheme and bufferless baseline system over multi-path Rayleigh fading channels. Based on the BER analysis, we further analyzed the LLR and channel capacity of the proposed system.
3. The theoretical results are corroborated by Monte Carlo simulations. Compared with existing RIS-aided DCSK-WPC, RM-DCSK-WPC, and the bufferless RIS-RM-DCSK-WPC benchmark systems, the proposed system achieves superior BER performance. Furthermore, the effects of several key system parameters on overall performance are evaluated.

The remainder of this paper is organized as follows. Section 2 introduces the system model of the proposed RIS-RM-DCSK-WPC. Section 3 provides a comprehensive performance analysis of the proposed system. Simulation results are reported in Section 4. Finally, Section 5 concludes this paper.

## 2. System and Signal Model

**Figure 1:**
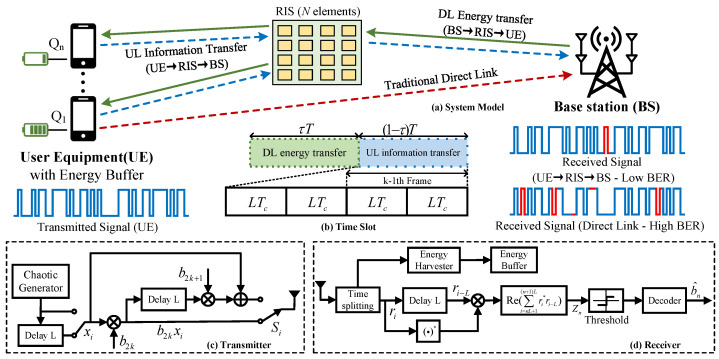
Block diagram of RIS-empowered RM-DCSK wireless powered communication system with energy-buffer.

In this paper, an RIS-RM-DCSK-WPC system model is proposed, which supports uplink data backhaul for energy-constrained wireless devices. Figure 1a shows that the considered model involves a base station (BS) with multiple antennas, an *N*-element RIS, and *Q* single-antenna user equipment (UE). Both the BS and the UE employ the RIS-RM-DCSK-WPC transmitter and receiver illustrated in Figure 1c,d.

To facilitate the analysis, we consider the downlink energy transfer (DL-ET) and uplink information transmission (UL-IT) processes over a representative single-link setup comprising a BS, an RIS, and a UE, and model the entire procedure as a normalized time slot-based system. The corresponding time-domain signal relationship is shown in Figure 1b. By adopting a time-switching protocol, the proposed transmission procedure consists of the following two phases.

In the DL-ET phase, the BS sends RF energy signals to the energy-limited UE via the RIS-empowered link. The chaotic signal generator at the BS transmitter produces 2β chaotic samples as the energy transfer signal, which occupies the first τT(0<τ<1) interval of the time slot.

In the UL-IT phase, the UE utilizes the harvested and stored energy to send data information back to BS through RIS-Empowered link. The data signal transmitted by the UE RM-DCSK transmitter consists of two equal time slots, in which the chaotic sequence transmitted in each slot is reused as the reference for the following slot while simultaneously conveying one information bit, and occupies the remaining (1−τ)T interval of the time slot system. The sequence length in the DL-ET phase is 2β, while the related sequence length in the UL-IT phase is 2L. The total sequence length within one time slot is denoted by $\beta_{T}$, i.e., $\beta_{T}=2\beta +2L$.

Specifically, during the first slot of the *k*-th frame, a chaotic signal $X_{k}$ of length *L* modulated by $b_{2k}\in \left\{-1,+1\right\}$ is transmitted. This transmission fulfills a dual role by conveying information and providing a reference for the subsequent slot of the same frame. In the second slot, the previously transmitted reference segment is further multiplied by another information bit $b_{2k+1}\in \left\{-1,+1\right\}$ and superimposed with the reference signal of the next frame, denoted by $X_{k+1}$. Consequently, the transmit signal of the *k*-th frame in the information transmission phase is given by

(1) $$S_{i}=\left\{\begin{array}{cc} b_{2k}x_{i} & 2kL<i\leq (2k+1)L\\ b_{2k+1}b_{2k}x_{i-L}+x_{i} & (2k+1)L<i\leq 2(k+1)L \end{array}\right.$$

where the chaotic sequence $x_{i}$ satisfies

(2) $$x_{(2k+1)N+n}=x_{2(k+1)N+n}\;\forall k\in \left\{0,\pm 1,\cdots \right\};0<n\leq N$$

As shown in Figure 1c,d, the transmitted waveform $S_{i}$ experiences multi-path fading and time splitting and results in the received signal $r_{i}$. The receiver then performs complex conjugation on $r_{i}$ and computes its correlation with a delayed replica of itself. Together with a conventional time slot synchronization circuit, the transmitted bit can be recovered. Hence, the correlator output $Z_{n}$ for recovering $b_{n}$ is given by

(3) $$Z_{n}=ℜ\left(\sum_{i=nL+1}^{(n+1)L}r_{i}^{*}\;r_{i-L}\right)$$

where ℜ(·) denotes the real-part operator and ${\left(\cdot \right)}^{*}$ is the conjugate operation.

The BS is assumed to be connected to a stable power supply, whereas UE has no fixed power source and must rely on harvested energy to support uplink communication. In the DL-ET phase, the BS transmits an RF signal to UE with a constant power $P_{BS}$. After going through RIS-Empowered channel, the signal received at UE, denoted by $r_{UE}$, can be expressed as

(4) $$\begin{array}{c} r_{UE}=\sqrt{P_{BS}}\sum_{l=1}^{L_{2}}\sum_{m=1}^{L_{1}}\left(\sum_{n=1}^{N}\varepsilon_{b,r}\varepsilon_{r,u}h_{n,m}\kappa_{n}e^{j\theta_{n}}w_{n,l}\right)\times S_{BS,i-\tau_{m}^{h}-\tau_{l}^{w}}+n_{UE,i} \end{array}$$

here $\varepsilon_{b,r}=\sqrt{d_{b,r}^{-\zeta_{b,r}}}$ and $\varepsilon_{r,u}=\sqrt{d_{r,u}^{-\zeta_{r,u}}}$ denote the path loss coefficients for the BS-RIS and RIS-UE links, determined by their respective distances $d_{b,r}$, $d_{r,u}$ and path loss exponent $\zeta_{b,r}$, $\zeta_{r,u}$. For the *n*-th RIS element, $h_{n,m}$ and $w_{n,l}$ denote the fading coefficients of the *m*-th BS-RIS path and *l*-th RIS-UE path, with delays $\tau_{m}^{h}$ and $\tau_{l}^{w}$, respectively. $n_{UE,i}$ models the additive white Gaussian noise (AWGN) following the distribution $n_{UE,i}∼\mathrm{CN}\left(0,N_{0}\right)$.

As illustrated in Figure 1d, the RM-DCSK receiver is fitted with an EH circuit to extract energy from the RF signal and store it for future use. Following common assumptions adopted in the literature [31], the BS-radiated RF signal is considered the sole energy supply for the UE, while the harvested energy is stored and becomes available only in later transmission intervals. Under this EH model, the energy harvested X(i) by the proposed system in time slot *i* is expressed as

(5) $$X\left(i\right)=\eta \tau P_{BS}h_{DL}\left(i\right),$$

where η(0<η<1) is the energy conversion efficiency factor. It should be noted that practical energy harvesting circuits are inherently nonlinear. The adopted linear model may therefore introduce two limitations: the neglect of saturation effects and the omission of the sensitivity threshold. Nevertheless, this linear approximation facilitates the derivation of a closed-form expression for the system BER. The detailed theoretical analysis is provided in Section 3.

Both the UL and DL links are modeled as flat block fading, where the channel coefficients remain constant within each time slot but vary independently across different slots. Thus, the DL channel power gain $h_{DL}\left(i\right)$ during the *i*-th time slot can be written as

(6) $$h_{DL}\left(i\right)=\sum_{l=1}^{L_{2}}\sum_{m=1}^{L_{1}}{\left|\sum_{n=1}^{N}\varepsilon_{b,r}\varepsilon_{r,u}h_{n,m}\left(i\right)w_{n,l}\left(i\right)\right|}^{2}$$

where $h_{DL}\left(i\right)$ and X(i) are independent stationary ergodic processes with mean $\Omega_{DL}=E\left[h_{DL}\left(i\right)\right]$ and $\bar{X}=\eta \tau P_{\mathrm{BS}}\Omega_{DL}$, respectively.

Considering the possible presence of obstacles on the communication link between BS and UE, the RIS illustrated in Figure 1a may not have access to the channel phase information $\theta_{n}$ or the amplitude reflection coefficients $\kappa_{n}$. Accordingly, a blind transmission scheme is adopted [29,32], in which the RIS passively reflects the incident signals without performing phase adjustment or any additional signal processing.

Compared with increasing the BS transmit power, which can only improve downlink energy harvesting in one direction and does not enhance uplink data transmission, blind RIS transmission can effectively overcome the path loss in both uplink and downlink. For active relay devices, blind RIS transmission is completely passive, and since it does not require dynamic phase controllers, its hardware and energy consumption costs are almost zero.

Specifically, the reflection phase of the RIS is defined as $\theta_{n}=0\;\left(n=1,2,\cdots ,N\right)$, and the amplitude reflection coefficient is fixed as $\kappa_{n}=1$, similar to the configurations considered in [29,30]. Hence, $r_{UE}$ can be further simplified as

(7) $$\begin{array}{c} r_{UE}=\sqrt{P_{BS}}\sum_{l=1}^{L_{2}}\sum_{m=1}^{L_{1}}\left(\sum_{n=1}^{N}\varepsilon_{b,r}\varepsilon_{r,u}h_{n,m}w_{n,l}\right)\times S_{BS,i-\tau_{m}^{h}-\tau_{l}^{w}}+n_{UE,i} \end{array}$$

Based on the above energy harvesting model and channel characteristics, and considering that accurate channel state information (CSI) is hard to estimate in practical scenarios, an on–off policy (OOP) [21] that does not require CSI is incorporated into the HSU framework to optimize the system performance. Specifically, a fixed threshold *B* is employed to regulate information transmission. During the data transmission phase of each time slot, UE transmits information with a predefined constant expected transmit power when the stored energy is sufficient; otherwise, the UE remains silent when the available energy cannot support data transmission. Thus, the transmit power of the UE is

(8) $$P_{UE}\left(i\right)=\left\{\begin{array}{cc} 0 & B(i)\leq B\\ B & B(i)>B \end{array}\right.$$

where B(i) is the energy stored in the energy buffer at the beginning of slot *i* and X(i) is accumulated in a finite-capacity *K* energy storage unit, such as a rechargeable battery. The evolution of the stored energy is is updated as follows

(9) $$\begin{array}{cc} B(i+1) & =\min\left(B\left(i\right)-P_{UE}\left(i\right)+X\left(i\right),K\right) \end{array}$$

where B(i) is a discrete-time Markov chain on a continuous state space *V*, where V=[0,K] for a finite-size energy buffer [13]. Therefore, the statistical behavior of the energy storage process can be characterized by the transition probability density function. Let $f_{X}\left(x\right)$ denote the PDF of the harvested energy X(i) within the *i*-th time slot. According to the on–off transmission policy described in (8) and (9), the one-step transition PDF p(x|u) from the current energy state B(i)=u to the next state B(i+1)=x can be expressed as

(10) $$p\left(x|u\right)=\left\{\begin{array}{cc} f_{X}\left(x-u\right), & u\leq B\\ f_{X}\left(x-u+B\right), & u>B \end{array}\right.$$

where 0⩽x<K. When the stored energy reaches the battery capacity limit *K*, probability mass accumulation occurs, and the corresponding transition probability is determined by the complementary cumulative distribution function (CCDF) of X(i). Accordingly, the steady-state distribution of the energy buffer, denoted by π(i), satisfies

(11) $$\pi \left(x\right)=\int_{0}^{B}\pi \left(u\right)f_{X}\left(x-u\right)du+\int_{B}^{K}\pi \left(u\right)f_{X}\left(x-u+B\right)du$$

In the UL-IT phase, the UE expends the energy accumulated in its storage unit to convey information to the BS over the RIS-aided uplink. Accordingly, the received signal at the BS can be written as

(12) $$\begin{array}{c} r_{BS}=\sqrt{P_{UE}}\sum_{l=1}^{L_{2}}\sum_{m=1}^{L_{1}}\left(\sum_{n=1}^{N}\varepsilon_{b,r}\varepsilon_{r,u}h_{n,m}w_{n,l}\right)\times S_{UE,i-\tau_{m}^{h}-\tau_{l}^{w}}+n_{BS,i} \end{array}$$

where $n_{BS,i}∼\mathrm{CN}\left(0,N_{0}\right)$ is AWGN.

## 3. Performance Analysis

### 3.1. BER Analysis

The chaotic sequence is generated by the second-order Chebyshev polynomial function (CPF) [33], i.e.,

(13) $$x_{i+1}=1-2x_{i}^{2}$$

where the mean $E[x_{i}]=0$ and variance $Var\left[x_{i}\right]=E\left[x_{i}^{2}\right]-E^{2}\left[x_{i}\right]=0.5$.

In this paper, it is assumed that the maximum multi-path delay $\tau_{m}^{h}$ and $\tau_{l}^{w}$ are much shorter than the symbol interval, i.e., $0<\tau_{m}^{h}\ll LT_{c}$, $0<\tau_{l}^{w}\ll LT_{c}$, such that the inter-symbol interference (ISI) can be neglected [5,6]. Moreover, for a sufficiently large spreading factor, the following approximation holds

(14) $$\sum_{i=(2k+1)L+1}^{2(k+1)L}x_{i-\tau_{l}}x_{i-\tau_{m}}\approx 0,\;l\neq m.$$

For the proposed system, it is assumed that perfect bit synchronization is achieved. The assumption is discussed in the simulation section. Thus, the outputs of the correlator for bits $b_{2k+1}$ and $b_{2k}$ are given, respectively, by

(15) $$\begin{array}{cc} \; & Z_{2k+1}\\ \; & \;=ℜ\left(\sum_{i=(2k+1)L+1}^{2(k+1)L}r_{\mathrm{BS},i}^{*}r_{\mathrm{BS},i-L}\right)\\ \; & \;=ℜ\left(\sum_{i=(2k+1)L+1}^{2(k+1)L}\left(\sqrt{P_{UE}}\sum_{l=1}^{L_{2}}\sum_{m=1}^{L_{1}}\left(\sum_{n=1}^{N}\varepsilon_{b,r}\varepsilon_{r,u}h_{n,m}w_{n,l}\right)\right.\right.\\ \; & \;\times S_{UE,i-\tau_{m}^{h}-\tau_{l}^{w}}+n_{\mathrm{BS},i}{)}^{*}\times \left(n_{\mathrm{BS},i-L}+S_{UE,i-\tau_{m}^{h}-\tau_{l}^{w}-L}\right.\\ \; & \;\times \sqrt{P_{UE}}\sum_{l=1}^{L_{2}}\sum_{m=1}^{L_{1}}\left(\sum_{n=1}^{N}\varepsilon_{b,r}\varepsilon_{r,u}h_{n,m}w_{n,l}\right))) \end{array}$$

(16) $$\begin{array}{cc} \; & Z_{2k}\\ \; & \;=ℜ\left(\sum_{i=2kL+1}^{(2k+1)L}r_{\mathrm{BS},i}^{*}r_{\mathrm{BS},i-L}\right)\\ \; & \;=ℜ\left(\sum_{i=2kL+1}^{(2k+1)L}\left(\sqrt{P_{UE}}\sum_{l=1}^{L_{2}}\sum_{m=1}^{L_{1}}\left(\sum_{n=1}^{N}\varepsilon_{b,r}\varepsilon_{r,u}h_{n,m}w_{n,l}\right)\right.\right.\\ \; & \;\times S_{UE,i-\tau_{m}^{h}-\tau_{l}^{w}}+n_{\mathrm{BS},i}{)}^{*}\times \left(n_{\mathrm{BS},i-L}+S_{UE,i-\tau_{m}^{h}-\tau_{l}^{w}-L}\right.\\ \; & \;\times \sqrt{P_{UE}}\sum_{l=1}^{L_{2}}\sum_{m=1}^{L_{1}}\left(\sum_{n=1}^{N}\varepsilon_{b,r}\varepsilon_{r,u}h_{n,m}w_{n,l}\right))) \end{array}$$

where

(17) $$\begin{array}{c} H_{i}=\sqrt{P_{UE}}\sum_{l=1}^{L_{2}}\sum_{m=1}^{L_{1}}\left(\sum_{n=1}^{N}\varepsilon_{b,r}\varepsilon_{r,u}h_{n,m}w_{n,l}\right)\times S_{UE,i-\tau_{m}^{h}-\tau_{l}^{w}}\\ H_{i-L}=\sqrt{P_{UE}}\sum_{l=1}^{L_{2}}\sum_{m=1}^{L_{1}}\left(\sum_{n=1}^{N}\varepsilon_{b,r}\varepsilon_{r,u}h_{n,m}w_{n,l}\right)\times S_{UE,i-\tau_{m}^{h}-\tau_{l}^{w}-L} \end{array}$$

The above formula can be further expressed as

(18) $$\begin{array}{cc} \; & Z_{2k+1}\\ \; & =\underset{U}{\underset{︸}{ℜ\left(\sum_{i=(2k+1)L+1}^{2(k+1)L}H_{i}^{*}H_{i-L}\right)}}+\underset{N_{1}}{\underset{︸}{ℜ\left(\sum_{i=(2k+1)L+1}^{2(k+1)L}n_{\mathrm{BS},i}^{*}n_{\mathrm{BS},i-L}\right)}}\\ \; & +\underset{N_{2}}{\underset{︸}{ℜ\left(\sum_{i=(2k+1)L+1}^{2(k+1)L}H_{i-L}n_{\mathrm{BS},i}^{*}\right)}}+\underset{N_{3}}{\underset{︸}{ℜ\left(\sum_{i=(2k+1)L+1}^{2(k+1)L}H_{i}^{*}n_{\mathrm{BS},i-L}\right)}} \end{array}$$

where *U* represents the useful signal component, and $N_{1}$, $N_{2}$, and $N_{3}$ represent the interference components generated by Gaussian noise.

Due to $n_{BS,i}$ following a complex Gaussian distribution, define $n_{BS,i}=a_{i}+jb_{i}$, $n_{BS,i-L}=a_{i-L}+jb_{i-L}$, where $a_{i},b_{i},a_{i-L},b_{i-L}∼\mathrm{CN}\left(0,\frac{N_{0}}{2}\right)$, then $N_{1}$ can be calculated as

(19) $$\begin{array}{c} N_{1}=\sum_{i=(2k+1)L+1}^{2(k+1)L}\left(a_{i}a_{i-L}+b_{i}b_{i-L}\right) \end{array}$$

Next, define $\sum_{n=1}^{N}\varepsilon_{b,r}\varepsilon_{r,u}h_{n,m}w_{n,l}=c+jd$. Then $N_{2}$ and $N_{3}$ can be expanded as

(20) $$\begin{array}{c} N_{2}=b_{2k}x_{i-L}\sqrt{P_{UE}}\sum_{i=(2k+1)L+1}^{2(k+1)L}\sum_{l=1}^{L_{2}}\sum_{m=1}^{L_{1}}\left(a_{i}c+b_{i}d\right) \end{array}$$

(21) $$\begin{array}{c} N_{3}=\sqrt{P_{UE}}\sum_{i=(2k+1)L+1}^{2(k+1)L}\sum_{l=1}^{L_{2}}\sum_{m=1}^{L_{1}}\left(a_{i-L}c+b_{i-L}d\right)\times \left(b_{2k+1}b_{2k}x_{i-L}+x_{i}\right) \end{array}$$

Given $b_{2k+1}=1$, the decision variable $Z_{2k+1}$ follows a Gaussian distribution. The mean and variance of *U*, $N_{1}$, $N_{2}$, and $N_{3}$ are respectively calculated as

(22) $$E\left[U\right]=\frac{P_{UE}L}{2}\sum_{l=1}^{L_{2}}\sum_{m=1}^{L_{1}}{\left|\sum_{n=1}^{N}\varepsilon_{b,r}\varepsilon_{r,u}h_{n,m}w_{n,l}\right|}^{2},\;Var\left[U\right]=0$$

(23) $$E\left[N_{1}\right]=0,\;Var\left[N_{1}\right]=\frac{LN_{0}^{2}}{2}$$

(24) $$E\left[N_{2}\right]=0,\;Var\left[N_{2}\right]=\frac{N_{0}P_{UE}L}{4}\sum_{l=1}^{L_{2}}\sum_{m=1}^{L_{1}}{\left|\sum_{n=1}^{N}\varepsilon_{b,r}\varepsilon_{r,u}h_{n,m}w_{n,l}\right|}^{2}$$

(25) $$E\left[N_{3}\right]=0,\;Var\left[N_{3}\right]=\frac{N_{0}P_{UE}L}{2}\sum_{l=1}^{L_{2}}\sum_{m=1}^{L_{1}}{\left|\sum_{n=1}^{N}\varepsilon_{b,r}\varepsilon_{r,u}h_{n,m}w_{n,l}\right|}^{2}$$

Hence, for $b_{2k+1}=1$, the mean and variance of $Z_{2k+1}$ can be written as, respectively,

(26) $$\begin{array}{cc} \; & E\left\{Z_{2k+1}|b_{2k+1}=1\right\}\\ \; & =E\left[U\right]+E\left[N_{1}\right]+E\left[N_{2}\right]+E\left[N_{3}\right]\\ \; & =\frac{P_{UE}L}{2}\sum_{l=1}^{L_{2}}\sum_{m=1}^{L_{1}}{\left|\sum_{n=1}^{N}\varepsilon_{b,r}\varepsilon_{r,u}h_{n,m}w_{n,l}\right|}^{2}\\ \; & \mathrm{Var}\left\{Z_{2k+1}|b_{2k+1}=1\right\}\\ \; & =\mathrm{Var}\left[U\right]+\mathrm{Var}\left[N_{1}\right]+\mathrm{Var}\left[N_{2}\right]+\mathrm{Var}\left[N_{3}\right]\\ \; & =\frac{3P_{UE}L}{4}\sum_{l=1}^{L_{2}}\sum_{m=1}^{L_{1}}{\left|\sum_{n=1}^{N}\varepsilon_{b,r}\varepsilon_{r,u}h_{n,m}w_{n,l}\right|}^{2}N_{0}+\frac{LN_{0}^{2}}{2} \end{array}$$

Similarly, for $b_{2k+1}=-1$, the mean and variance of $Z_{2k+1}$ are given, respectively, by

(27) $$\begin{array}{cc} \; & E\left\{Z_{2k+1}|b_{2k+1}=-1\right\}=\frac{-P_{UE}L}{2}\sum_{l=1}^{L_{2}}\sum_{m=1}^{L_{1}}{\left|\sum_{n=1}^{N}\varepsilon_{b,r}\varepsilon_{r,u}h_{n,m}w_{n,l}\right|}^{2}\\ \; & \mathrm{Var}\left\{Z_{2k+1}|b_{2k+1}=-1\right\}=\frac{3P_{UE}L}{4}\sum_{l=1}^{L_{2}}\sum_{m=1}^{L_{1}}{\left|\sum_{n=1}^{N}\varepsilon_{b,r}\varepsilon_{r,u}h_{n,m}w_{n,l}\right|}^{2}N_{0}+\frac{LN_{0}^{2}}{2} \end{array}$$

Based on (26) and (27), the Gaussian approximation method is utilized here, then the BER for the $b_{2k+1}$ can be described as

(28) $$\begin{array}{cc} \; & {\mathrm{BER}}_{b_{2k+1}}\\ \; & =\frac{1}{2}P\left(Z_{2k+1}<0|b_{2k+1}=1\right)+\frac{1}{2}P\left(Z_{2k+1}>0|b_{2k+1}=-1\right)\\ \; & =\frac{1}{2}\mathrm{erfc}\left(\frac{E\left\{Z_{2k+1}|b_{2k+1}=1\right\}}{\sqrt{2\mathrm{Var}\left\{Z_{2k+1}|b_{2k+1}=1\right\}}}\right)\\ \; & =\frac{1}{2}\mathrm{erfc}\left({\left(\frac{6}{L\gamma_{\mathrm{BS}}}+\frac{4}{L\gamma_{\mathrm{BS}}^{2}}\right)}^{-\frac{1}{2}}\right)\\ \; & =\frac{1}{2}\mathrm{erfc}\left({\left(\frac{4}{\left(1-\tau \right)\beta_{t}\gamma_{\mathrm{BS}}}\left(3+\frac{2}{\gamma_{\mathrm{BS}}}\right)\right)}^{-\frac{1}{2}}\right) \end{array}$$

A similar conclusion applies to the recovery of the bit $b_{2k}$. Then, the BER performance of the uplink can be written as

(29) $${\mathrm{BER}}_{UL}=\frac{1}{2}\mathrm{erfc}\left({\left(\frac{4}{\left(1-\tau \right)\beta_{t}\gamma_{\mathrm{BS}}}\left(3+\frac{2}{\gamma_{\mathrm{BS}}}\right)\right)}^{-\frac{1}{2}}\right)$$

where erfc(·) represents the complementary error function, and $\gamma_{BS}$ is the SNR of the UE-BS, i.e.,

(30) $$\gamma_{\mathrm{BS}}=\frac{P_{UE}\sum_{l=1}^{L_{2}}\sum_{m=1}^{L_{1}}{\left|\sum_{n=1}^{N}\varepsilon_{b,r}\varepsilon_{r,u}h_{n,m}w_{n,l}\right|}^{2}}{N_{0}}$$

Without loss of generality, the channel gains of each link are assumed to be uniformly distributed [29,30], i.e., $E\left[{\left|h_{n,1}\right|}^{2}\right]=\cdots =E\left[{\left|h_{n,m}\right|}^{2}\right]$, $E\left[{\left|w_{n,1}\right|}^{2}\right]=\cdots =E\left[{\left|w_{n,m}\right|}^{2}\right]$.This assumption can obtain the upper bound of the BER [6]. Furthermore, normalize the channel coefficients *h* and *w* of the uplink and downlink, where $h∼\mathrm{CN}\left(0,1\right)$, $w∼\mathrm{CN}\left(0,1\right)$, $h_{n,m}∼\mathrm{CN}\left(0,1/L_{1}\right)$, and $w_{n,l}∼\mathrm{CN}\left(0,1/L_{1}\right)$. Define $\lambda =\sum_{n=1}^{N}\varepsilon_{b,r}\varepsilon_{r,u}h_{n,m}w_{n,l}$, $H=\sum_{l=1}^{L_{2}}\sum_{m=1}^{L_{1}}{\left|\lambda \right|}^{2}$. For a sufficiently large number of reflecting elements, i.e., N≫1, according to the central limit theorem (CLT) [34], $\lambda ∼\mathrm{CN}\left(0,\frac{{\left(\varepsilon_{b,r}\varepsilon_{r,u}\right)}^{2}N}{L_{1}L_{2}}\right)$. It follows that ${\left|\lambda \right|}^{2}∼\chi^{2}\left(2\right)$ and $H∼\chi^{2}\left(2L_{1}L_{2}\right)$. Consequently, the probability density function of *H* is

(31) $$\begin{array}{c} f_{H}\left(x\right)=\frac{x^{L_{1}L_{2}-1}}{\Gamma \left(L_{1}L_{2}\right){\left(\frac{{\left(\varepsilon_{b,r}\varepsilon_{r,u}\right)}^{2}N}{L_{1}L_{2}}\right)}^{L_{1}L_{2}}}\times \exp\left(-\frac{xL_{1}L_{2}}{{\left(\varepsilon_{b,r}\varepsilon_{r,u}\right)}^{2}N}\right) \end{array}$$

Given that the transmit power $P_{UE}$ remains constant within a single time slot, the BER expression for the proposed system is given by

(32) $$\begin{array}{cc} \; & {\mathrm{BER}}_{HSU}\\ \; & =\int_{0}^{\infty }\frac{1}{2}\mathrm{erfc}\left({\left(\frac{4}{\left(1-\tau \right)\beta_{t}\gamma_{\mathrm{BS}}}\left(3+\frac{2}{\gamma_{\mathrm{BS}}}\right)\right)}^{-\frac{1}{2}}\right)f_{H}\left(x\right)dx\\ \; & =\int_{0}^{\infty }\frac{1}{2}\mathrm{erfc}\left({\left(\frac{4}{\left(1-\tau \right)\beta_{t}\gamma_{\mathrm{BS}}}\left(3+\frac{2}{\gamma_{\mathrm{BS}}}\right)\right)}^{-\frac{1}{2}}\right)\\ \; & \;\times \frac{x^{L_{1}L_{2}-1}}{\Gamma \left(L_{1}L_{2}\right){\left(\frac{{\left(\varepsilon_{b,r}\varepsilon_{r,u}\right)}^{2}N}{L_{1}L_{2}}\right)}^{L_{1}L_{2}}}\exp\left(-\frac{xL_{1}L_{2}}{{\left(\varepsilon_{b,r}\varepsilon_{r,u}\right)}^{2}N}\right)dx \end{array}$$

For the bufferless RIS-RM-DCSK-WPC system, all energy harvested at the UE is immediately consumed for data transmission in the current time slot, i.e., harvest-use (HU) protocol. The transmit power $P_{UE}$ is expressed as

(33) $${P_{UE}}^{\prime }=\frac{\eta P_{\mathrm{BS}}h_{DL}\tau }{1-\tau }$$

Its probability density function is

(34) $$\begin{array}{c} f_{{P_{UE}}^{\prime }}\left(x\right)=\frac{x^{L_{1}L_{2}-1}}{\left(L_{1}L_{2}-1\right)!{\left(\frac{\eta P_{\mathrm{BS}}\tau {\left(\varepsilon_{b,r}\varepsilon_{r,u}\right)}^{2}N}{\left(1-\tau \right)L_{1}L_{2}}\right)}^{L_{1}L_{2}}}\times \exp\left(-\frac{x\left(1-\tau \right)L_{1}L_{2}}{\eta P_{\mathrm{BS}}\tau {\left(\varepsilon_{b,r}\varepsilon_{r,u}\right)}^{2}N}\right) \end{array}$$

Thus, the BER formula of the bufferless RIS-RM-DCSK-WPC system is

(35) $${\mathrm{BER}}_{HU}=\int_{0}^{\infty }\frac{1}{2}\mathrm{erfc}\left({\left(\frac{6}{L\gamma_{\mathrm{BS}}}+\frac{4}{L\gamma_{\mathrm{BS}}^{2}}\right)}^{-\frac{1}{2}}\right)f_{{P_{UE}}^{\prime }}\left(x\right)dx$$

### 3.2. LLR Derivation and Information-Theoretic Capacity Analysis

This section analyzes the LLR of the modulated bits and the equivalent channel capacity of the proposed system over Rayleigh fading channels. Based on the previous BER analysis, for a sufficiently large spreading factor *L*, the decision variables $Z_{2k+1}$ and $Z_{2k}$ can be approximated as Gaussian random variables, and the ISI can be neglected. Assuming that the transmitted bits at the transmitter are equiprobable, the conditional probability density functions (PDFs) of the decision statistics are given by $p\left(Z_{2k+1}|b_{2k+1}=-1\right)∼N\left(-\mu_{Z},\sigma_{Z}^{2}\right)$ and $p\left(Z_{2k+1}|b_{2k+1}=+1\right)∼N\left(\mu_{Z},\sigma_{Z}^{2}\right)$. According to (26) and (27), conditioned on the channel state *H*, the conditional mean $\mu_{Z}$ and variance $\sigma_{Z}^{2}$ can be expressed as

(36) $$\begin{array}{cc} \; & \mu_{Z}=\frac{P_{UE}L}{2}\sum_{l=1}^{L_{2}}\sum_{m=1}^{L_{1}}{\left|\sum_{n=1}^{N}\varepsilon_{b,r}\varepsilon_{r,u}h_{n,m}w_{n,l}\right|}^{2}\\ \; & \sigma_{Z}^{2}=\frac{3P_{UE}L}{4}\sum_{l=1}^{L_{2}}\sum_{m=1}^{L_{1}}{\left|\sum_{n=1}^{N}\varepsilon_{b,r}\varepsilon_{r,u}h_{n,m}w_{n,l}\right|}^{2}N_{0}+\frac{LN_{0}^{2}}{2} \end{array}$$

Therefore, the expression for the LLR of the modulated bit $b_{2k+1}$ is given by

(37) $$\begin{array}{c} L\left(Z_{2k+1}\right)=\log\frac{p\left(Z_{2k+1}|b_{2k+1}=+1\right)}{p\left(Z_{2k+1}|b_{2k+1}=-1\right)}=\log\frac{\exp\left(-\frac{{\left(Z_{2k+1}-\mu_{Z}\right)}^{2}}{2\sigma_{Z}^{2}}\right)}{\exp\left(-\frac{{\left(Z_{2k+1}+\mu_{Z}\right)}^{2}}{2\sigma_{Z}^{2}}\right)}=\frac{2\mu_{Z}}{\sigma_{Z}^{2}}Z_{2k+1} \end{array}$$

Specifically, the above LLR expression can further support the implementation of soft-decision decoding for channel coding.

Furthermore, the proposed system is designed and analyzed without requiring CSI. From an information-theoretic perspective, its receiver employs hard-decision demodulation, which aligns with the standard demodulation scheme adopted by most DCSK receivers. To simplify the analysis, we abstract the entire transmission link of the system as an equivalent Binary Symmetric Channel (BSC). The previously derived theoretical BER can directly serve as the crossover probability of this equivalent channel, denoted as $P_{e}$. Thus, the equivalent channel capacity can be formulated as

(38) $$C=1+P_{e}\log_{2}\left(P_{e}\right)+\left(1-P_{e}\right)\log_{2}\left(1-P_{e}\right)$$

It is worth noting that the analysis presented in this section extends the preceding BER derivations into the information-theoretic domain. The derived LLR expression provides the necessary input for soft-decision decoding in potential coded system implementations, while the equivalent BSC channel capacity quantifies the fundamental information transmission capability of the system. These results demonstrate that the analytical framework established in this paper not only characterizes the fundamental performance of the uncoded system, but also lays the theoretical foundation for the design and optimization of subsequent channel coding systems.

### 3.3. Transmission Rate and Energy Efficiency Analysis

A comparison with the conventional DCSK scheme is carried out to evaluate the transmission rate and energy efficiency of RM-DCSK. The data rate improvement of RM-DCSK over DCSK is quantified by the percentage increase $R_{I}$, i.e.,

(39) $$R_{I}=\frac{R_{b,\mathrm{RM}-\mathrm{DCSK}}-R_{b,\mathrm{DCSK}}}{R_{b,\mathrm{DCSK}}}\times 100\%$$

where $R_{b,\mathrm{RM}-\mathrm{DCSK}}=\frac{1}{\beta_{1}}$ and $R_{b,\mathrm{DCSK}}=\frac{1}{2\beta_{1}}$ denote the data rate of RM-DCSK and DCSK, respectively, which can be simplified as

(40) $$R_{I}=\left(\frac{1/\beta_{1}}{1/2\beta_{1}}-1\right)\times 100\%=100\%$$

Consequently, given the same spreading factor $\beta_{1}$, the RM-DCSK system achieves a 100% increase in the data transmission rate compared to DCSK.

Let $S_{E}$ denote the energy-saving ratio of the RM-DCSK scheme relative to the conventional DCSK

(41) $$S_{E}=\frac{E_{b,\mathrm{RM}-\mathrm{DCSK}}-E_{b,\mathrm{DCSK}}}{E_{b,\mathrm{DCSK}}}\times 100\%$$

where $E_{b,\mathrm{RM}-\mathrm{DCSK}}=\frac{3\beta_{1}E\left[x_{k}^{2}\right]T_{c}}{2}$, $E_{b,\mathrm{DCSK}}=2\beta_{1}E\left[x_{k}^{2}\right]T_{c}$ represent the transmission energy per bit of RM-DCSK and DCSK, respectively, which can be further simplified as

(42) $$S_{E}=\left(1-\frac{3\beta_{1}E\left[x_{k}^{2}\right]T_{c}}{4\beta_{1}E\left[x_{k}^{2}\right]T_{c}}\right)\times 100\%=25\%$$

This indicates that the RM-DCSK system exhibits a superior energy efficiency comparison with DCSK, achieving 25% energy savings for each transmitted bit.

### 3.4. Complexity Analysis

In this subsection, the complexity of the proposed RIS-RM-DCSK-WPC system is evaluated against conventional DCSK and RM-DCSK. We analyze the complexity by counting the number of spreading and despreading operations at the transmitter and receiver [35]. For a fair comparison, all systems are assumed to employ the same spreading factor *L*.

In DCSK, one bit is transmitted per symbol period. The transmitter performs one spreading operation and the receiver performs one despreading operation. Therefore, the total number of operations required to transmit *M* bits is approximately 2M. For the RM-DCSK, two bits are conveyed per symbol period. The transmitter performs two spreading operations and one superposition, while the receiver performs two despreading operations. Although the per symbol operations double, the information per symbol also doubles, resulting in unchanged per bit complexity. Thus, the total number of operations required to transmit *M* bits is approximately 5M/2.

For the proposed RIS-RM-DCSK-WPC system, the transmitter complexity is the same as RM-DCSK since the RIS is deployed in the channel. The receiver complexity remains the same as in the other two systems. Moreover, the energy management in WPC only involves a threshold comparison with complexity O(1). The RIS operates in a blind transmission mode without phase optimization or channel estimation, thereby introducing only O(1) computational complexity.

Consequently, the proposed RIS-RM-DCSK-WPC system achieves a 100% spectral efficiency improvement and a 25% energy efficiency gain at the cost of only a marginal complexity increase (an additional 0.5 operation per bit at the transmitter and no increase at the receiver).

## 4. Performance Evaluation of RIS-RM-DCSK-WPC

In this section, the BER performance of the proposed RIS-RM-DCSK-WPC system is evaluated under multi-path Rayleigh fading channels. Without loss of generality, the energy storage capacity *K* and the energy threshold *B* are fixed at 1 J and 0.25 J, respectively. A dual-path Rayleigh fading channel is considered, with path loss coefficients set as follows: $E\left[{\left|h_{n,1}\right|}^{2}\right]=E\left[{\left|h_{n,2}\right|}^{2}\right]=\frac{1}{2}$, $\tau_{1}^{h}=0$, $\tau_{2}^{h}=1$; $E\left[{\left|w_{n,1}\right|}^{2}\right]=E\left[{\left|w_{n,2}\right|}^{2}\right]=\frac{1}{2}$, $\tau_{1}^{w}=0$, $\tau_{2}^{w}=2$, $d_{b,r}=1$ m, $d_{r,u}=10$ m, and $\varepsilon_{b,r}=\varepsilon_{r,u}=2$. The total spread factor $\beta_{T}$ is set to 640, while the energy harvesting time factor τ and energy conversion efficiency factor η are set to 0.6 and 0.7, respectively.

### 4.1. BER Performance Comparison Between Analytical and Simulation Results

**Figure 2:**
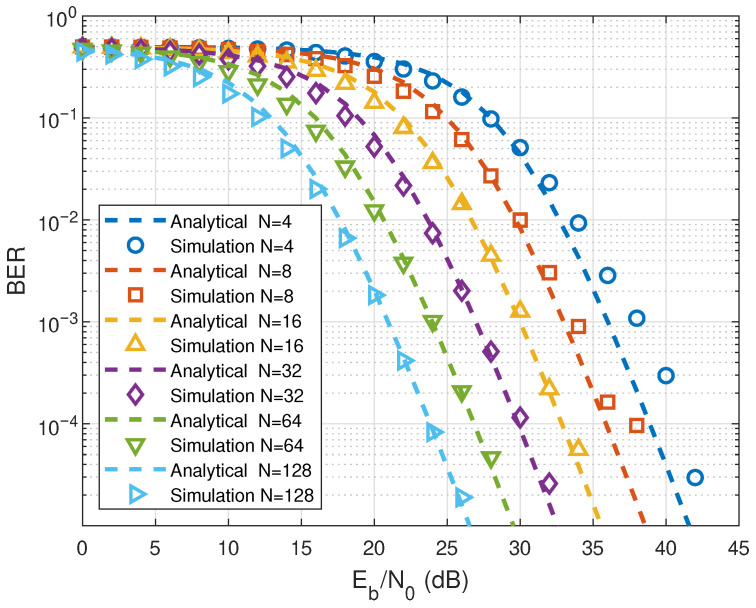
Comparison between analytical and simulated BER curves of the proposed RIS-RM-DCSK-WPC system for different numbers of *N*.

Numerical simulations are provided to corroborate the analytical BER performance derived for the proposed system. Figure 2 illustrates the BER performance of the proposed RIS-RM-DCSK-WPC system with different RIS element *N* values. As can be perceived in Figure 2, the analytical results are well validated by the simulation results, which validates the correctness of the previously proposed analysis. It is further observed that increasing *N* significantly improves the BER performance of the proposed system, as all BER curves shift downward with larger N under the same $E_{b}/N_{0}$. For a BER of $10^{-4}$, the proposed system achieves approximately 3 dB performance gain with *N* = 128 compared to N=64, and approximately 6 dB performance gain compared to N=32. In addition, it indicates that the interference limit resulting from the reference reuse structure will not restrict the BER performance under high SNR conditions.

**Figure 3:**
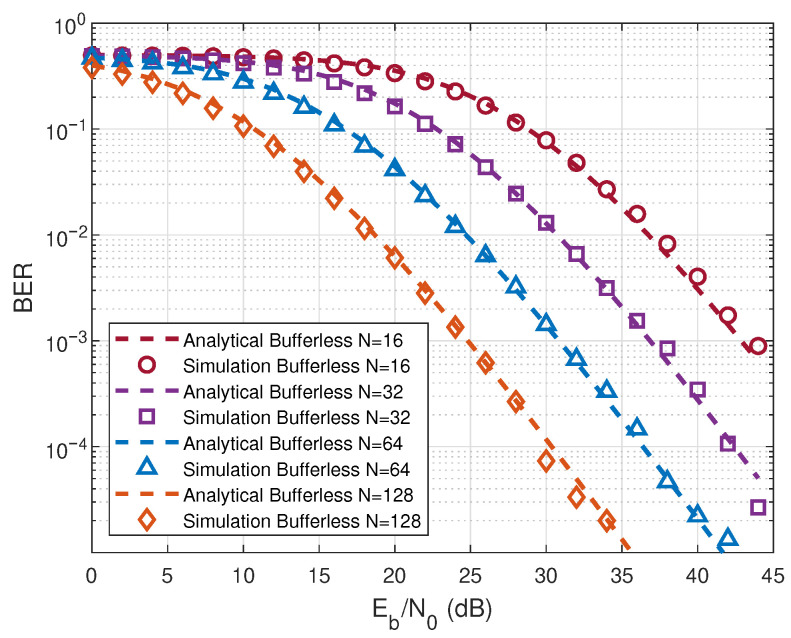
BER performance of the energy bufferless RIS-RM-DCSK-WPC system for different values of *N*.

Figure 3 illustrates the analytical and simulated BERs of the energy bufferless RIS-RM-DCSK-WPC system with different *N* values. In Figure 3, the analytical results of the energy bufferless RIS-RM-DCSK-WPC system align with the simulation results, validating the correctness of the previously proposed analysis. Specifically, the case with N=128 achieves the lowest BER among the considered configurations, whereas N=16 exhibits the worst error performance, indicating that a larger number of reflecting elements provides a higher effective reflecting gain and thus enhances the received SNR. Moreover, the BER improvement becomes more evident in the moderate-to-high $E_{b}/N_{0}$, where the performance gap between different *N* values widens, highlighting the impact of RIS on reliability. From Figure 2 and Figure 3, it can be seen that both the proposed system and the other bufferless system achieve optimal BER performance at N=128.

### 4.2. BER Performance Comparison with Benchmark Systems

**Figure 4:**
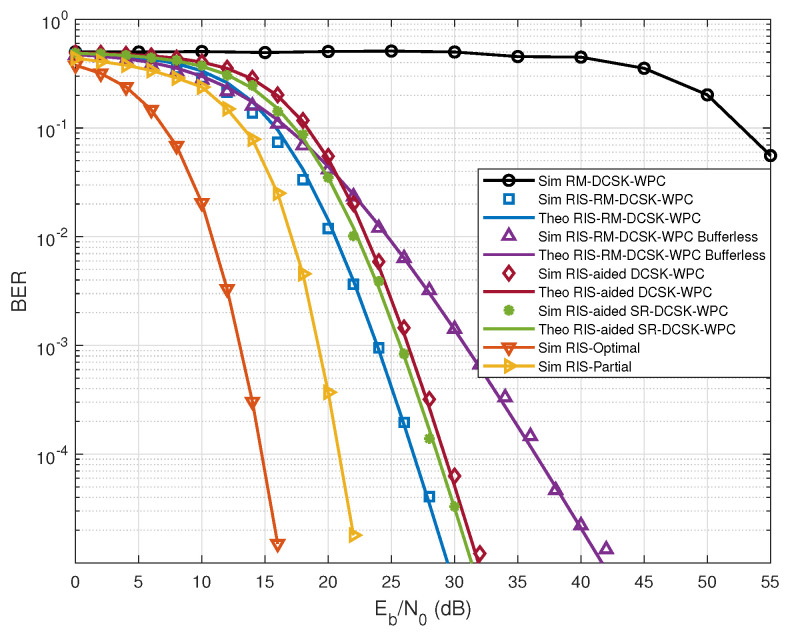
BER results for the proposed RIS-RM-DCSK-WPC and other systems under N=64, $\beta_{T}=640$, K=1 J, and B=0.25 J.

To validate the effectiveness of the proposed scheme, we present a comparative BER analysis against benchmark systems. Figure 4 plots the BER curves of the proposed RIS-RM-DCSK-WPC and the benchmark systems with N=64 under multi-path Rayleigh fading channels. As shown in Figure 4, the proposed scheme achieves substantial BER performance gains over RM-DCSK-WPC system, which is because the RIS can adjust the wireless signal propagation environment to enhance BER performance. It is worth noting that the simulation results indicate that the proposed system achieves superior BER performance compared with the RIS-aided DCSK-WPC [29], the bufferless RIS-RM-DCSK-WPC, and the recently proposed RIS-aided SR-DCSK-WPC scheme [30]. These results clearly verify the enhanced reliability of the proposed system. In addition, we further compare the proposed scheme with both ideally phase-optimized RIS and partially optimized RIS configurations. It can be seen that the ideal phase optimization achieves the best performance, while the partial optimization effectively reduces the performance gap between the blind scheme and the ideal case. Nevertheless, both optimized RIS schemes rely on a complex channel estimator to acquire CSI, leading to increased implementation complexity.

**Figure 5:**
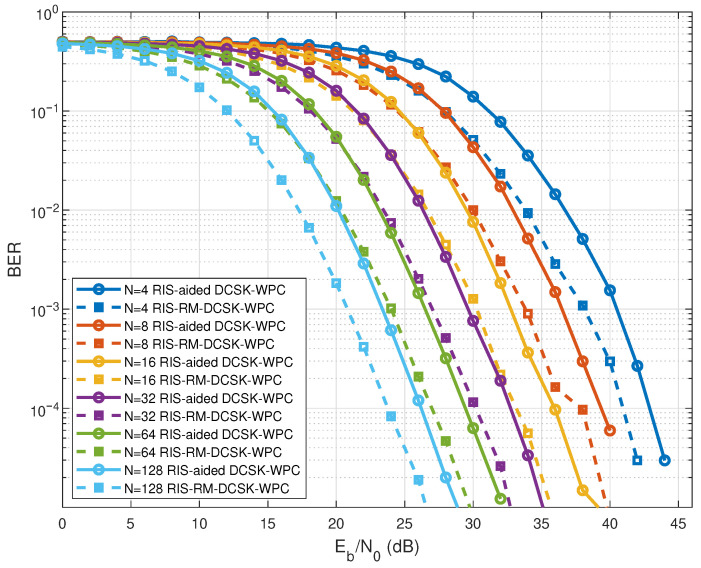
BER performance of RIS-RM-DCSK-WPC and RIS-aided DCSK-WPC for different numbers of *N* with K=1 J and B=0.25 J.

To highlight the performance advantage of the proposed system under varying RIS reflecting elements *N*, Figure 5 shows a detailed BER performance comparison between the proposed scheme and the existing RIS-aided DCSK-WPC system for varying numbers of *N*. Moreover, the proposed RIS-RMDCSK-WPC scheme consistently outperforms the RIS-aided DCSK-WPC benchmark for all the considered *N* values, indicating that the performance gain is not limited to a specific RIS size. As can be observed, for a BER level of $10^{-4}$, the proposed system provides an SNR improvement of about 3 dB compared with the RIS-aided DCSK-WPC scheme.

**Figure 6:**
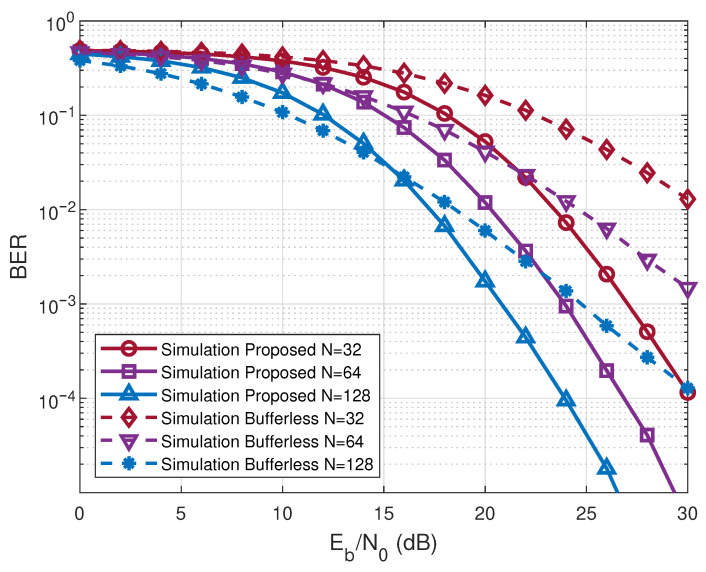
BER performance comparison between the proposed system and the bufferless RIS-RM-DCSK-WPC system for different numbers of *N*.

Figure 6 plots the BER curves of the proposed scheme and the bufferless RIS-RM-DCSK-WPC system. As is shown in Figure 6, the proposed system consistently achieves a lower BER compared to its non-buffered counterpart with the SNR increases. This is primarily because the limitations of the HU protocol employed by the non-buffered system, where communication reliability is strictly constrained by the instantaneous level of harvested energy. In contrast, the proposed system utilizes a HSU mechanism integrated with an energy management strategy, avoiding energy waste and BER problems caused by insufficient energy.

### 4.3. Parameters and Imperfect Bit Synchronization Analysis

**Figure 7:**
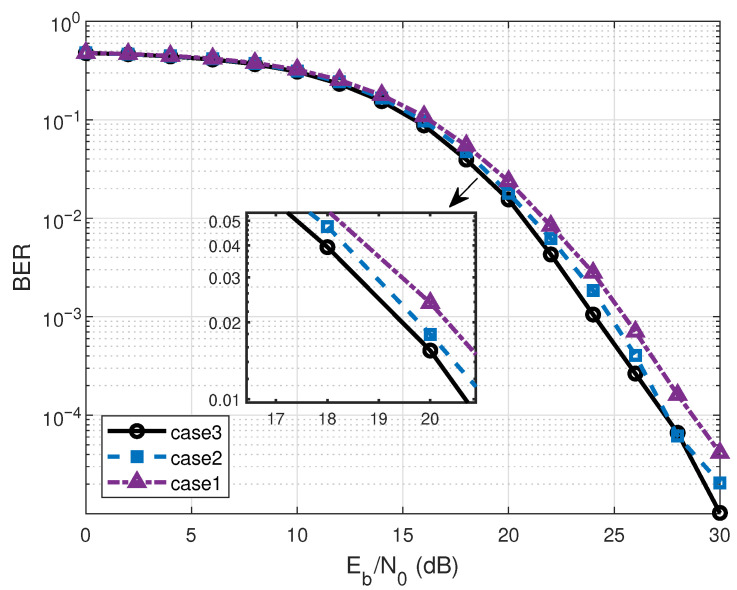
BER performance of the proposed system over three different channel gains for N=64.

To provide further insights into the system design, the following numerical results illustrate the effects of critical parameters on the BER and energy behavior of the proposed RIS-RM-DCSK system. Figure 7 shows the BER performance of the proposed system over different channel gains, where channel gains are given in Table 1. For fairness, the propagation delays of all channels are set to the same values. The results demonstrate that equal channel gains yield the best BER performance for the proposed system. It is worth noting that all simulation experiments in this work are conducted under the assumption of equal channel gains to ascertain the optimal BER performance.

**Figure 8:**
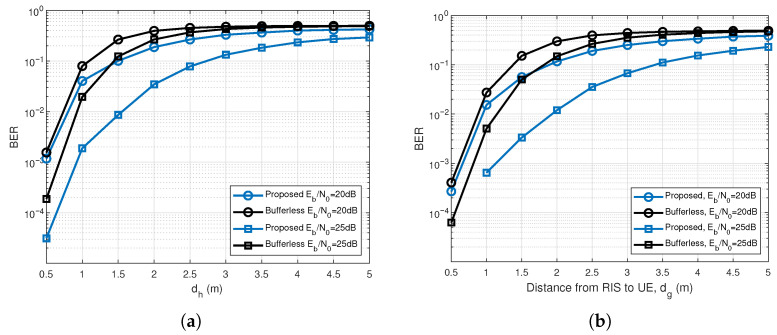
BER performance comparison of the proposed system and the bufferless RIS-RM-DCSK-WPC system versus the BS–RIS distance and the RIS–UE distance for N=64. (**a**) BER versus the BS–RIS distance. (**b**) BER versus the RIS–UE distance.

Figure 8 plots the BER curves of the proposed scheme and its bufferless counterpart versus the BS–RIS distance and the RIS–UE distance for N=64. Owing to the reliance on both uplink and downlink transmissions, the RIS placement, the deployment position of RIS is directly related to the total path loss. As shown in Figure 8a,b, the BER is extremely sensitive to the position of RIS. Shortening the BS–RIS or RIS–UE distance consistently improves the BER performance. Specifically, when the RIS is deployed within 3 m of either the BS or the UE, the presence of the RIS has a pronounced impact on the system performance. This confirms that deploying the RIS in close proximity to the BS or UE effectively mitigates path loss.

**Figure 9:**
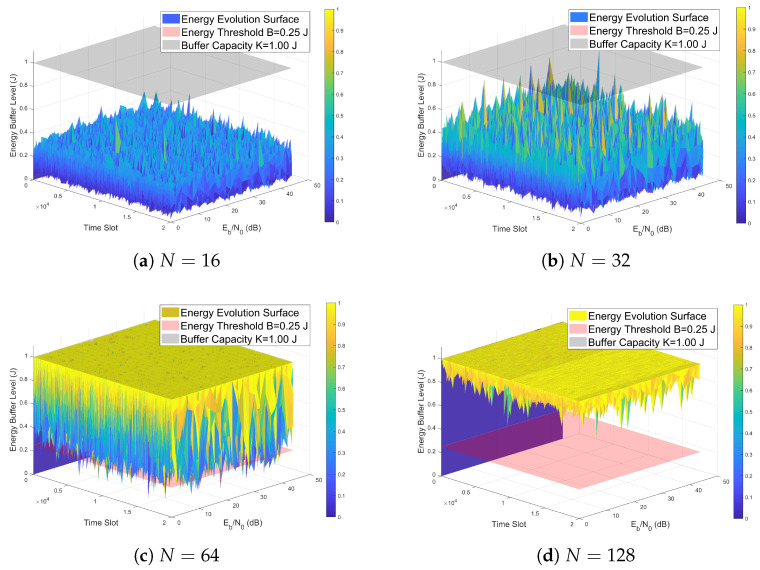
Evolution of stored energy level in the proposed system over varying *N*. (**a**) N=16. (**b**) N=32. (**c**) N=64. (**d**) N=128.

Figure 9 illustrates the dynamic variations of the energy buffer in the proposed system. According to the multi-path fading nature of the channel, the harvested energy displays random fluctuations. When the energy stored in the buffer drops below the threshold *B*, the user equipment enters a silent energy harvesting phase, resulting in a temporary suspension of data transmission. Conversely, once the accumulated energy exceeds *B*, the user equipment immediately resumes data transmission at a fixed power level, meanwhile consuming the stored energy.

**Figure 10:**
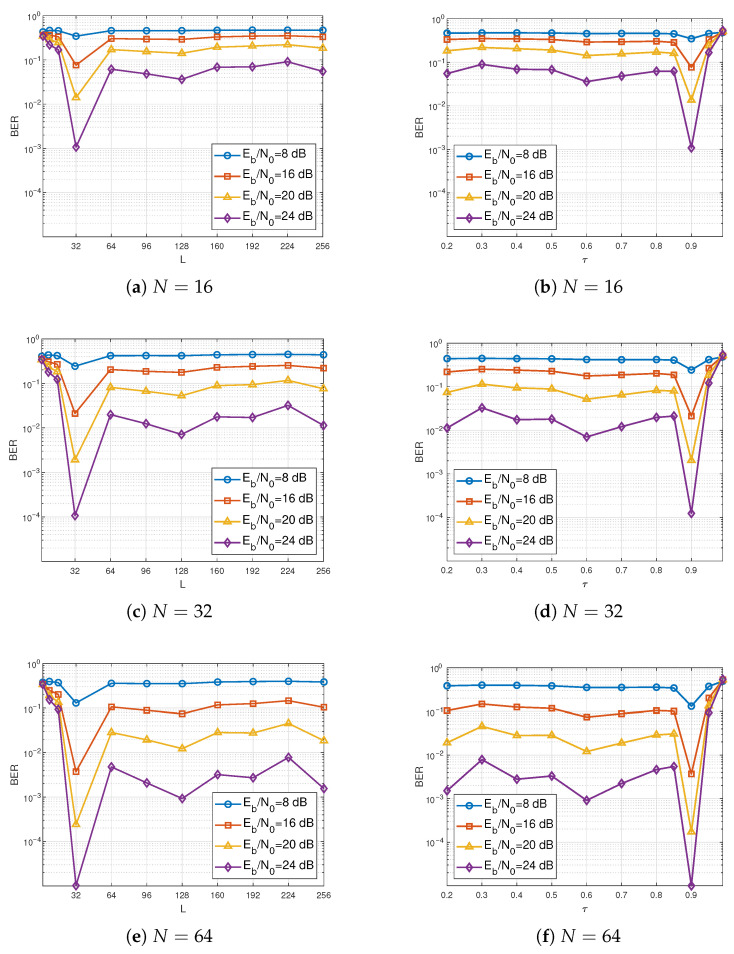
BER performance of the proposed system versus the spreading factor *L* and the energy harvesting time factor τ under different $E_{b}/N_{0}$ values. (**a**,**c**,**e**) present the effect of *L* for N=16, N=32, and N=64, respectively, whereas (**b**,**d**,**f**) depict the impact of τ for the same values of *N*.

We evaluate the influence of the spreading factor and energy harvesting time factor on the BER performance of the proposed scheme. Figure 10 presents the relationship between BER and the spreading factor and the energy harvesting time factor over different $E_{b}/N_{0}$, respectively. As shown in Figure 10, the system achieves the optimal BER performance at L=32, regardless of the values of *N* or $E_{b}/N_{0}$. Furthermore, the optimal BER performance is achieved when τ=0.9, which is consistent with the previous analysis. Specifically, according to $L=\left(1-\tau \right)\beta_{T}/2$, having $\beta_{T}$ as 640 and τ as 0.9 yields L=32, corresponding to the best BER performance.

**Figure 11:**
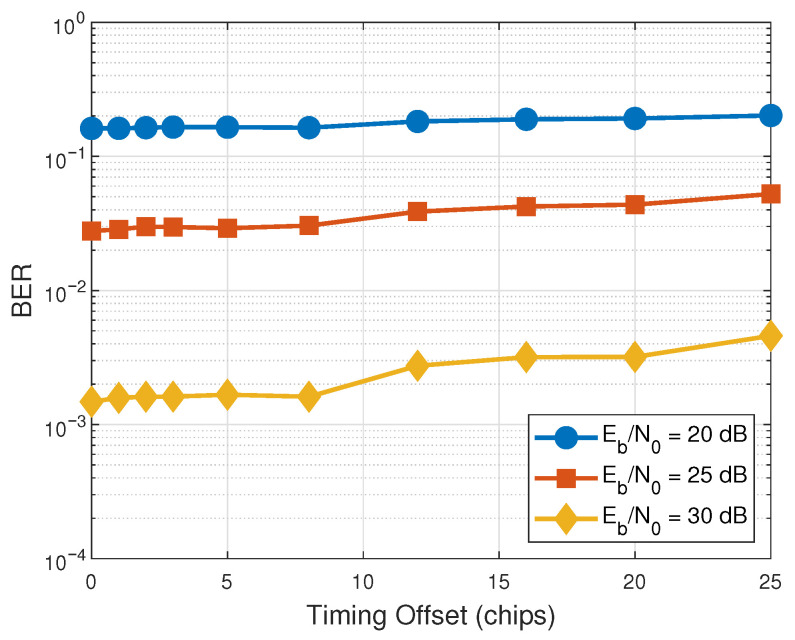
BER performance versus timing offset for the proposed RIS-RM-DCSK-WPC system under different $E_{b}/N_{0}$ levels.

In the theoretical analysis presented in Section 3, perfect bit synchronization is assumed. We evaluate the system’s sensitivity to imperfect bit synchronization. Figure 11 illustrates the BER performance of the proposed RIS-RM-DCSK-WPC system versus the timing offset under different $E_{b}/N_{0}$ levels (20 dB, 25 dB, and 30 dB). It is clearly observed that the BER performance exhibits strong robustness to minor synchronization errors. Specifically, for a timing offset between 0 and 8 chips, the BER degradation is almost negligible across all $E_{b}/N_{0}$ levels. The performance only begins to degrade marginally when the offset exceeds 10 chips. Thus, the simulation has confirmed that the perfect bit synchronization assumption we adopted in our theoretical derivation is reasonable.

### 4.4. Throughput Analysis

**Figure 12:**
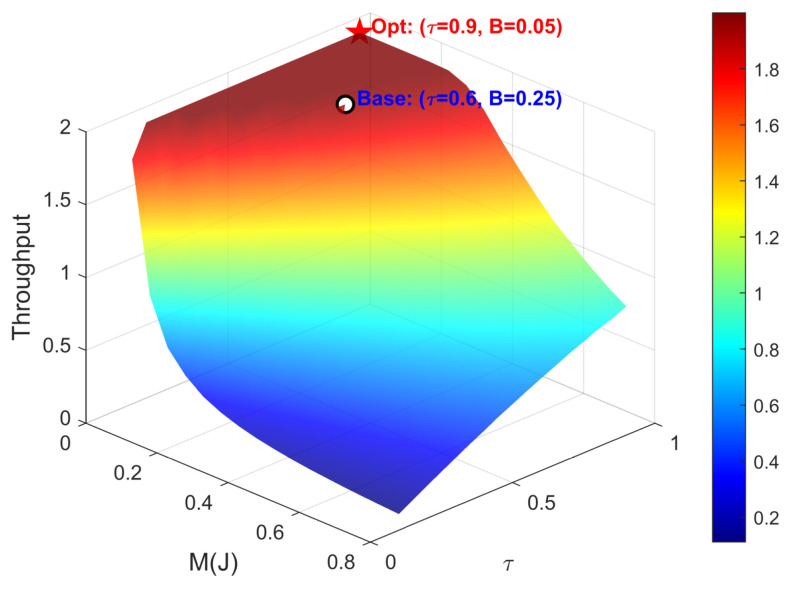
The variation of system throughput with τ and *B* at $E_{b}/N_{0}=30$ dB.

To comprehensively evaluate the system performance, we explore maximizing the normalized throughput T by jointly optimizing τ and *B*. The throughput is defined as the average number of successfully transmitted effective bits per unit time $T\left(\tau ,B\right)=\frac{2}{T}\cdot P_{tx}\left(\tau ,B\right)\cdot \left[1-\mathrm{BER}\left(\tau ,B\right)\right]$. This optimization problem faces a core physical trade-off: increasing τ can improve the transmission probability ($P_{tx}$), but it will compress the information transmission time and thus increase the bit error rate (BER); increasing *B* can enhance the transmit power to reduce the BER, but it will increase the energy outage probability and thus lead to a decrease in $P_{tx}$. Figure 12 plots the 3D throughput surface at $E_{b}/N_{0}=30$ dB. As theoretically anticipated, the surface exhibits pronounced non-convexity with a unique global maximum. Compared with the baseline throughput of 1.9523 (bps/Hz), the optimized design increases the throughput to 1.9993 (bps/Hz), yielding a 2.41% gain.

## 5. Conclusions

In this paper, an RIS-empowered reference-modulated differential chaos shift keying wireless powered communication (RIS-RM-DCSK-WPC) system for energy-constrained uplink data reporting is proposed. By jointly introducing RIS-aided propagation reconfiguration and the RM-DCSK scheme, the proposed system effectively mitigates severe path loss impairments and improves spectral efficiency while retaining the key benefits of noncoherent communication, including low circuit complexity and no channel state information (CSI) requirement. Closed-form BER expressions are derived over multi-path Rayleigh fading channels, and the theoretical analyses are validated through Monte Carlo simulations, and comparative evaluations demonstrate that the proposed system achieves superior BER performance compared with existing RIS-aided DCSK-WPC and a bufferless benchmark system. These results indicate that the proposed system is a promising candidate for low-cost, low-power, and high-throughput wireless communication. In our future work, we will extend the proposed single-link architecture to a multi-user framework, with a primary focus on mitigating multi-user interference (MUI). Furthermore, it is of great significance to conduct further research on RIS phase optimization, as well as on the coded performance and coding optimization based on the proposed system.

## Figures and Tables

**Table 1:** Channel gain coefficients.

Channel	$E\left[{\left|h_{n,1}\right|}^{2}\right]$	$E\left[{\left|h_{n,2}\right|}^{2}\right]$	$E\left[{\left|w_{n,1}\right|}^{2}\right]$	$E\left[{\left|w_{n,2}\right|}^{2}\right]$
Case1	1/4	3/4	2/9	7/9
Case2	1/3	2/3	3/10	7/10
Case3	1/2	1/2	1/2	1/2

## Data Availability

Data is contained within the article.
